# 鼻甲血管内大B细胞淋巴瘤伴噬血细胞淋巴组织细胞增生症1例报告并文献复习

**DOI:** 10.3760/cma.j.cn121090-20250303-00108

**Published:** 2025-11

**Authors:** 馨予 张, 璐 何, 淑颖 马, 燕平 刘, 重阳 丁, 磊 范, 建勇 李, 祎 缪

**Affiliations:** 1 南京医科大学第一附属医院（江苏省人民医院）血液科，南京 210029 Department of Hematology, the First Affiliated Hospital of Nanjing Medical University（Jiangsu Province Hospital）, Nanjing 210029, China; 2 南京大学医学院附属鼓楼医院病理科，南京 210028 Department of Pathology, the Affiliated Nanjing Drum Tower Hospital, Nanjing University Medical School, Nanjing 210028, China; 3 南京医科大学第一附属医院（江苏省人民医院）病理科，南京 210029 Department of Pathology, the First Affiliated Hospital of Nanjing Medical University（Jiangsu Province Hospital）, Nanjing 210029, China; 4 南京医科大学第一附属医院（江苏省人民医院）核医学科，南京 210029 Department of nuclear medicine, the First Affiliated Hospital of Nanjing Medical University（Jiangsu Province Hospital）, Nanjing 210029, China

## Abstract

血管内大B细胞淋巴瘤（IVLBCL）是弥漫大B细胞淋巴瘤的一种罕见亚型，预后不良。鼻甲是IVLBCL极为罕见的受累部位。本文报道1例51岁女性，主诉不明原因发热1个月，实验室检查显示血细胞减少、高甘油三酯血症、铁蛋白水平升高、可溶性CD25升高以及骨髓中存在噬血细胞现象；未发现明显感染证据；PET-CT显示鼻甲摄取^18^F-氟代脱氧葡萄糖（FDG）异常。鼻甲活检组织学显示肿瘤细胞主要位于血管腔内，表达CD20、BCL6、PAX5、MUM1，Ki-67>60％。因此，诊断为IVLBCL伴有噬血细胞淋巴组织细胞增生症（HLH）。患者接受1个周期DEP（脂质体多柔比星+依托泊苷+甲泼尼龙）方案控制HLH，随后接受5个周期R-CHOP（利妥昔单抗+环磷酰胺+多柔比星+长春新碱+泼尼松）方案化疗和自体造血干细胞移植（auto-HSCT）巩固治疗，病情持续缓解。IVLBCL的预后各异，早期诊断和及时治疗可能与长期生存相关，R-CHOP方案联合auto-HSCT治疗有效。

## Abstract (en)

Intravascular large B cell lymphoma（IVLBCL）is a rare, aggressive subtype of diffuse large B cell lymphoma, with nasal turbinate involvement being uncommon. We report a 51-year-old woman with a 1-month history of fever of unknown origin. Laboratory findings showed cytopenia, hypertriglyceridemia, elevated ferritin, increased soluble CD25, and bone marrow hemophagocytosis. No infectious cause was identified. PET-CT revealed abnormal ^18^F-fluorodeoxyglucose（FDG）uptake in the nasal turbinates. Turbinate biopsy revealed tumor cells localized predominantly within vascular lumens, positive for CD20, BCL6, PAX5, and MUM1, with a Ki-67 index >60％, confirming a diagnosis of IVLBCL with hemophagocytic lymphohistiocytosis（HLH）. The patient received one cycle of the DEP regimen（liposomal doxorubicin, etoposide, and methylprednisolone）for HLH, followed by five cycles of R-CHOP（rituximab, cyclophosphamide, doxorubicin, vincristine, and prednisone）and consolidation with auto-HSCT, achieving sustained complete remission. IVLBCL outcomes are heterogeneous; early diagnosis and prompt treatment improve survival, and R-CHOP plus auto-HSCT may be an effective strategy.

血管内大B细胞淋巴瘤（IVLBCL）是一种罕见的非霍奇金淋巴瘤亚型，发病率低于1％，其特征是大量肿瘤细胞积聚在小或中等大小血管腔内，特别是毛细血管和后毛细血管微血管。最常见的受累部位是中枢神经系统（CNS）和皮肤，也可累及肺、肾、肝、骨髓及其他结外部位[1]。根据肿瘤细胞浸润部位的不同，IVLBCL临床表现各异。目前有两种常见类型：经典型或“西方型”，在欧洲人中更常见，以受累器官的临床症状为特征，皮肤和CNS受累常见；“亚洲型”或噬血细胞综合征相关型，在亚洲人中更常见，以噬血细胞性淋巴组织细胞增生症（HLH）、骨髓受累、B症状（发热、盗汗、体重减轻）、低蛋白血症、肝脾肿大和血细胞减少为特征[2]。多样且非特异性的临床表现使得IVLBCL的早期诊断和治疗十分困难，导致疾病预后不良。病理组织学检查仍是确诊IVLBCL的重要方法。IVLBCL患者的预后差，中位生存期为1年[3]。由于IVLBCL的罕见性，对该疾病的认识并不统一，诊断与治疗方面缺乏规范化的共识与策略。本文描述了1例鼻甲IVLBCL合并HLH的诊疗过程并进行相关文献复习。

## 病例资料

患者，51岁女性，因“不明原因高热1个月”于2020年8月就诊南京医科大学第一附属医院。体格检查未发现明显异常。实验室检查异常值包括：血红蛋白65 g/L，血小板84×10^9^/L，白蛋白25.5 g/L，乳酸脱氢酶823 U/L，甘油三酯3.03 mmol/L，铁蛋白2 349.5 ng/ml，纤维蛋白原4.75 g/L，D-二聚体8.49 mg/L，可溶性CD25（sCD25）66 410 ng/L，红细胞沉降率58 mm/h，C反应蛋白186 mg/L，抗核抗体1∶320，降钙素原2.31 ng/ml，白细胞介素-6为469.42 pg/ml，白细胞介素-10为19 681.97 pg/ml，脑利钠肽前体417.9 pg/ml。患者进行感染相关检查，包括结核感染T细胞、病毒性肝炎、人类免疫缺陷病毒、梅毒、巨细胞病毒、EB病毒以及真菌检查（包括曲霉菌抗原和β-D-葡聚糖检测），结果均为阴性。抗生素、糖皮质激素和免疫抑制剂治疗无效，患者仍反复高热。PET-CT显示双侧中下鼻甲增厚，^18^F-氟代脱氧葡萄糖（FDG）摄取增高（SUVmax为8.1）。脾、肝和骨髓中也观察到弥漫性^18^F-FDG摄取增高（SUVmax为7.1）。随后，患者接受下、中鼻甲活检手术。显微镜下，肿瘤性淋巴细胞主要位于血管腔内，细胞质丰富，胞核不规则（图1）。免疫组织化学染色显示肿瘤细胞CD20、BCL6、PAX5、MUM1阳性，CD3、CD5、CD10、CD30、CD38、CD56、TIA-1、颗粒酶B、c-Myc、P53、EBNA-2、CK-pan、EBER阴性，Ki-67>60％（图2）。骨髓穿刺及活检显示三系造血正常，但组织细胞显著增加。对患者骨髓及鼻甲活检组织DNA进行二代基因检测，共检出体细胞变异62个，累及基因包括：ARID1A、BCL11A、BCL7A、BCLA2、BTG1、CD79B、CDK12、CIITA、CTNNB1、DUSP2、ERBB4、ETV6、FAT1、GRHPR、H1-2、H1-4、IGLL5、IRF4、ITPKB、KLHL14、KMT2D、MPEG1、MYD88、NF1、NSD2、OSBPL10、PIM1、RET、SETD1B、SETD2、SMAD4、TET3、TOX、TRAF3、TSC1、TUBB3、VMP1、ZBTB7A。基因重排1个：CD274::RHEX。患者诊断为IVLBCL伴有HLH（满足八项诊断标准中的七项：两系及以上血细胞减少、发热、脾肿大、高甘油三酯血症、铁蛋白水平升高、高sCD25和骨髓中噬血细胞现象）。给予患者1个周期DEP（脂质体多柔比星+依托泊苷+甲泼尼龙）方案治疗后患者体温控制，随后给予5个周期R-CHOP（利妥昔单抗+环磷酰胺+多柔比星+长春新碱+泼尼松）方案治疗并进行自体造血干细胞移植（auto-HSCT）作为巩固治疗。治疗结束时，复查PET-CT和骨髓穿刺及活检，患者达到完全缓解。目前已随访51个月，患者无复发迹象。

**图1:**
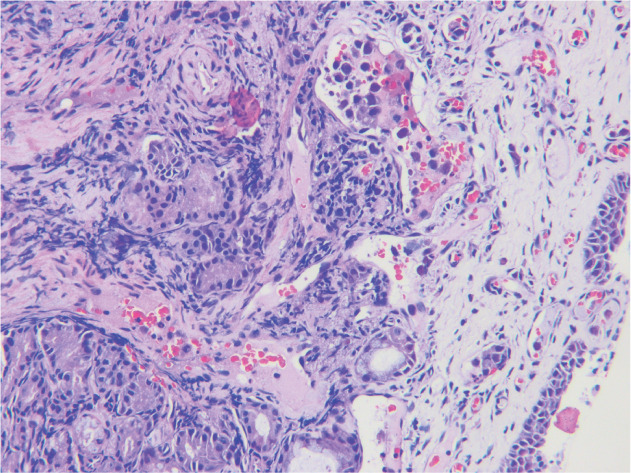
鼻甲血管内大B细胞淋巴瘤伴噬血细胞淋巴组织细胞增生症患者中、下鼻甲活检病理（HE染色，×200） **注** 非典型肿瘤细胞发生变形，聚集于血管腔内

**图2:**
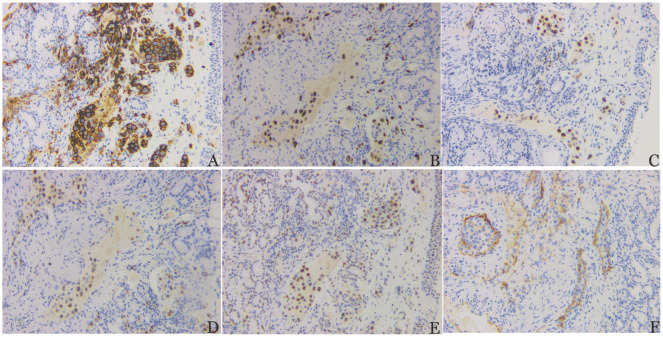
鼻甲血管内大B细胞淋巴瘤伴噬血细胞淋巴组织细胞增生症患者中、下鼻甲活检病理（免疫组织化学染色，×200） **A** CD20染色突显血管腔内非典型肿瘤细胞；**B** Ki-67阳性率高；**C～E** 肿瘤细胞PAX5、MUM1以及BCL6阳性；**F** CD10阴性

## 讨论及文献复习

IVLBCL是弥漫大B细胞淋巴瘤（DLBCL）的一种罕见亚型，占所有淋巴瘤的比例<1％，主要发生在中老年人。肿瘤细胞常侵犯各器官的小血管和毛细血管。由于其临床表现缺乏特异性，且较少表现为血管外肿块，因此IVLBCL难以诊断。患者通常有长期不明原因的发热、体重减轻或全身多系统炎症表现，大多数亚洲型患者有HLH和骨髓侵犯，还可能导致肾病综合征、高血压和全血细胞减少。IVLBCL是一种高度侵袭性淋巴瘤，对化疗反应差，死亡率高，早期诊断和治疗可能会改善患者预后。尽管目前尚无最佳诊断方案，但对于怀疑IVLBCL的患者，随机皮肤活检仍然是最常见的策略。PET-CT引导组织活检对于早期诊断IVLBCL也起到非常重要的作用。因此，对于那些怀疑IVLBCL且没有明显皮肤受累的患者，PET-CT可以作为皮肤活检前的监测工具[2],[4]。

亚洲型IVLBCL伴HLH的发生率明显高于西方型，为20％～60％[5]。继发于IVLBCL的HLH患者往往有更严重的临床症状，但对总生存的影响尚无定论。来自日本的一项大型回顾性数据中，伴或不伴有HLH的IVLBCL非影响生存的预后因素[6]。然而，部分研究发现伴有HLH的患者生存期明显短于不伴有HLH的患者[7]。

前期研究报道，CNS受累在西方型中比在亚洲型更常见。初诊时25％～50％的患者CNS受累，3年CNS复发风险为25％[8]–[9]。接受含或不含利妥昔单抗化疗的患者在CNS复发风险方面无显著差异[8]。伴有CNS受累的IVLBCL与疾病快速进展及不良预后相关[10]。CNS复发后，2年生存率仅为12％[8]。能穿透血脑屏障的药物是CNS淋巴瘤治疗的重要组成部分，但在CNS受累的IVLBCL中，甲氨蝶呤（MTX）等血脑屏障穿透药物并未获得更高的反应率和更长的生存期[3]。研究人员已经尝试了各种CNS预防策略，但目前为止，仍未在DLBCL中出现令人信服的疗效证据。此外，许多医疗中心采用鞘内预防，但也无法在CNS实质内达到足够的药物浓度[11]–[12]。众多研究包含部分前瞻性研究和荟萃分析也未能证明大剂量MTX和鞘内预防可降低CNS复发风险[13]–[14]。而对于IVLBCL这一特殊类型DLBCL，有研究表明，大剂量MTX有助于降低CNS复发[9]。而本例患者未接受CNS预防治疗。

R-CHOP方案是IVLBCL的一线治疗方案。尽管联合利妥昔单抗改善了患者预后[6],[15]，但患者的长期生存仍然不佳，中位生存期不到1年[3]。研究人员尝试通过更强化的治疗和auto-HSCT来克服不良预后。研究表明，大剂量化疗后进行auto-HSCT作为巩固治疗可能对IVLBCL有效，3年总生存率高达90％[16]。在利妥昔单抗时代，日本一项大样本研究以auto-HSCT作为一线巩固治疗，5年无进展生存率为80％[17]。基于这些研究，auto-HSCT可能是IVLBCL患者的一种选择。然而，auto-HSCT后仍有复发风险，目前没有足够的数据支持异基因造血干细胞移植（allo-HSCT）的疗效。只有少数报道auto-HSCT后复发的IVLBCL患者通过allo-HSCT成功治愈[18]。

在IVLBCL突变谱中，B细胞受体（BCR）/核因子κB（NF-κB）信号通路相关基因如MYD88、CD79B、IRF4等具有较高的突变频率[19]。本例患者同样检测到了这类突变。布鲁顿酪氨酸激酶（BTK）抑制剂对伴有MYD88和CD79B突变的B细胞淋巴瘤有效，而这类突变在IVLBCL中十分常见，提示IVLBCL可能从BTK抑制剂中获益。北京协和医院开展的一项前瞻性、单臂、Ⅱ期临床试验验证泽布替尼联合R-CHOP方案治疗初诊IVLBCL的疗效及安全性[20]。

本文报道了1例罕见的鼻甲受累的IVLBCL病例，该例患者的长期生存可能与疾病的早期诊断和治疗有关。R-CHOP联合auto-HSCT可以作为该疾病的有效治疗方法。由于缺乏大规模的前瞻性研究，有关该疾病的可用信息主要依赖于有限的病例报告，现有数据并不能证明IVLBCL的遗传背景与预后之间存在显著相关性。新的遗传发现可能是未来靶向治疗和临床试验的基础。未来仍需进一步的研究来提高对该疾病的认识并确定最佳治疗方法。
